# Functional synergistic effects of graphene nanoribbons and surfactant stabilizers on inhibition of growth of biofilm-forming and biofilm non-forming bacteria

**DOI:** 10.1007/s11356-026-37570-w

**Published:** 2026-03-27

**Authors:** Iaroslav Rybkin, Olga Zakharova, Alexander Gusev, Ales Lapanje

**Affiliations:** 1 Jozef Stefan Institute, Department of Environmental Sciences, Ljubljana, 1000 Slovenia; 2 Research Institute for Environmental Science and Biotechnology, Derzhavin Tambov State University, Tambov, 392000 Russia; 3 National University of Science and Technology MISIS, Moscow, 119991 Russia

**Keywords:** Graphene nanoribbons, Surfactants, Bactericidal activity, Biofilms, Pathogenic bacteria

## Abstract

In this study, we investigated the antimicrobial properties of graphene nanoribbons (GNRs) synthesized via a bottom-up approach. Due to their tendency to aggregate in aqueous solutions, various surfactants were used as stabilizers. We examined the effects of GNRs in combination with commonly used surfactants—including the cationic CTAB, anionic TWEEN, and non-ionic TRITON—to evaluate their impact on GNR toxicity. A range of GNR-surfactant concentrations was tested against biofilm-forming (*Escherichia coli* MG1655 and *Staphylococcus epidermidis* DSM 20044) and non-biofilm-forming (*E. coli* TOP10 and *S. epidermidis* BH1) bacterial strains under short-term (acute) and long-term (continuous) exposure conditions. CTAB alone exhibited antibacterial effects, but a synergistic interaction between CTAB and GNRs was observed during continuous exposure, particularly against Gram-positive bacteria. The capability to form biofilms did not significantly contribute to bacterial resistance, except in *E. coli* MG1655, which survived at the highest concentrations of GNR-CTAB during short-term exposure. In contrast to GNR-CTAB, GNR-TWEEN, and GNR-TRITON suspensions showed no inhibitory effects on bacterial growth and, in some cases, even promoted bacterial growth. Microscopic analysis revealed bacterial cell aggregation exclusively in GNR-CTAB suspensions. These findings highlight the critical role of surfactant selection in modulating GNR toxicity and provide insights into optimizing GNRs as antibacterial agents or minimizing their environmental impact.

**Supplementary Information:**

The online version contains supplementary material available at 10.1007/s11356-026-37570-w.

## Introduction

Carbon nanomaterials, including graphene nanoribbons (GNRs), are promising nanomaterials with a wide range of potential applications in biomedicine, biotechnology, agriculture, and electronics. GNRs are narrow strips of graphene that are one atomic layer thick and less than 50 nm wide. Their unique electrical, optical, and mechanical properties have attracted enormous interdisciplinary interest. GNRs have the potential to be used in a variety of devices and applications, including optoelectronic devices (Fu et al. 2018; Miao et al. 2021; Wieland et al. 2021), transparent conductive films (Kim et al. 2011; Ansón-Casaos et al. 2015), electronics (Chae and Lee 2014; Peng et al. 2019; Sengupta 2020), agriculture (Zaytseva and Neumann 2016), sensors (Shekhirev et al. 2020), DNA sequencing devices (Paulechka et al. 2016), drug and gene delivery systems (Chowdhury et al. 2015; Foreman et al. 2017), and tissue engineering scaffolds (Silva et al. 2017; Vasconcellos et al. 2021; Zakharova et al. 2021).

Given the potential for widespread use of graphene nanoribbons (GNRs) across various applications, it is essential to determine any adverse effects on microorganisms since they represent a critical component of the ecosystem. Yet, they play a crucial role in maintaining ecological balance as they are fundamental to nutrient cycling, organic matter decomposition, and the overall functioning of ecosystems. From a medical point of view, a better understanding of the antimicrobial action of GNRs could enable their safe use in medical applications, where the antimicrobial properties of GNRs hold promise as a potential alternative for infection treatment (Gungordu Er et al. 2023). In this regard, although a limited amount of information on GNRs toxic activity is reported, there are indications of their antibacterial activity against medically important strains (Ricci et al. 2017; Qiang et al. 2021). Currently it is known that GNRs can kill bacteria by damaging cellular components such as cell wall, proteins, lipids, and nucleic acids (reviewed by Zou et al. 2016). The antibacterial activity of graphene-like materials is explained primarily by the direct interaction between the nanomaterial and bacteria. In particular, graphene-like materials may interact with macromolecular components of the bacterial cell via hydrogen bonds, electrostatic adsorption, and π–π stacking affecting microbial viability. These interactions by accumulation of graphene-like particles on the cell surface result in chemical and structural changes of proteins, lipids, DNA/RNA as well as causing membrane damage and oxidative stress (Jarosz et al. 2016; Zhang et al. 2016; Mohammed et al. 2020; Frontiñan-Rubio et al. 2022; Majumder and Gangopadhyay 2022). Thus, GNRs have significant potential to exhibit antibacterial effects that require further study, especially in terms of factors that enhance or weaken their antimicrobial activity.

Since the GNRs are engineered carbon nanostructures with very low variability in characteristics due to precise control during synthesis (Narita et al. 2015; Zhang et al. 2015; Sun et al. 2020; Ma and Feng 2024), they represent one of the basic nanostructures that can be then further modified and adjusted for particular applications. Modifications to GNR introduce changes in both (i) intrinsic properties, such as lateral size, number of layers, shape, surface modifications, and agglomeration, and (ii) extrinsic properties, which arise from the stabilization of suspensions for various applications. For antimicrobial testing, GNRs must be stabilized in aqueous suspensions, as this is the primary condition under which antimicrobial activity can be evaluated in culture media that support microbial growth (Zou et al. 2016). Hence, each modification of the GNR changes the aggregation-dispersion properties in solvents (Piao et al. 2024), and various surfactants are usually added. Therefore, the toxicity of GNR can be weakened or enhanced based on the type and concentrations of the surfactants as well as the medium used in toxicity tests (Hui et al. 2014; Zou et al. 2016; Gusev et al. 2019a, 2019b; Pulingam et al. 2021; Piao et al. 2024). Media containing various nutrients to sustain microbial growth introduce additional factors contributing to antimicrobial activity, and the results cannot be directly deduced from the currently available information. For example, contrary to expectations, reduced graphene oxide rGO (Gusev et al. 2019a) as well as graphene oxide (GO) (Gusev et al. 2019b) were found to be more toxic to *E. coli* in a medium than in a saline solution. Moreover, the physiological microbial properties such as structure and stability of the cell wall (Rastogi et al. 1981; Dik et al. 2018; Nikolic and Mudgil 2023), neutralizing ROS formation (Lekmeechai et al. 2018), and the capability of formation of the biofilms (Angelini et al. 2023) can help bacteria to overcome the stress (Storz et al. 1990; Lushchak 2001). In particular, the formation of biofilms enables cells to become isolated from toxic substances or nanomaterials (Stewart 2002).

Moreover, as for many other nanomaterials, the main challenge in processing graphene-like materials is to prevent the formation of agglomerates (Alexander et al. 2021). The elevated specific surface area of graphene ribbons and their van der Waals interactions lead to a marked tendency to form irreversible aggregations or even convert into graphite. These latter materials have properties that are inferior in quality relative to graphene-like materials, as most of the prominent properties arise from their being composed of separate sheets (Chen et al. 2012). Such aggregation reduces the accessible surface area of graphene nanoribbons and compromises the physicochemical properties that are critical for their biological and antimicrobial activity. Achieving a homogeneous dispersion of GNRs is a critical issue due to graphene’s poor stability in most solvents. Good graphene dispersion within an aqueous media is also related to its stability over time and the ability of the media to prevent the graphene sheets from restacking (Nazari et al. 2019).

Given the complex toxic effects of nanomaterials on bacteria, our aim was to assess the antimicrobial activity of graphene nanoribbons by examining their interactions with different types of surfactants used for stabilization, as well as their effects on bacterial cell wall architecture and biofilm-forming capabilities. Specifically, we selected two types of surfactants: (i) a cationic surfactant known for its antimicrobial activity, and (ii) a surfactant without any reported toxicity to elucidate synergistic actions. Additionally, considering the tendency of graphene-based materials to attach to bacterial surfaces (Gusev et al. 2019a, 2019b) and therefore the expected immediate acute effect, here we also tested continuous exposure to observe potential long-term effects especially on cells that are more resistant to short exposures.

## Materials and methods

### Bacterial strains and growth conditions

In our study, we used four bacterial strains, two Gram-positive and two Gram-negative. The strain *Escherichia coli* MG 1655 (Gram-negative) and *Staphylococcus epidermidis* DSM 20044 (Gram-positive) are known to form biofilms, while *E. coli* TOP 10 (Gram-negative) and *S. epidermidis* BH1 (Gram-positive) are not capable of biofilm formation. The bacterial cells were prepared by transferring a single colony into 50 mL of fresh Nutrient Broth 1 medium (NB) (Carl Roth, cat. no. AE92.2) and incubating at 37 °C on a rotary shaker (Multitron, Infors) at 160 rpm till the cells reached stationary phase, optical density at 600 nm (OD_600nm_) ~ 1.2–1.5. The 30 mL of grown cultures were centrifuged for 5 min at 5000 g and washed 3 times in 30 mL sterile 0.9% NaCl. Finally, OD_600nm_ of each individual strain was adjusted to 0.1 for further experiments in a 200 μL volume within 96-well microtiter plate (VWR, cat. no. 734–2781).

### GNRs synthesis and characterization

The GNRs used in this study were provided and characterized by the group of Prof. Alexander Sinitskii (University of Nebraska-Lincoln, USA). They were synthesized via a well-established “bottom-up” approach based on the polymerization of molecular precursors using a Ni^0^-mediated Yamamoto coupling, followed by Scholl cyclodehydrogenation with iron(III) chloride, as described in detail previously (Vo et al. 2014). GNRs were characterized by transmission electron microscopy (TEM), Raman spectroscopy, and X-ray photoelectron spectroscopy (XPS). According to the published, the GNRs are predominantly monolayer (~ 0.3 nm thickness), with widths from several nanometers up to 60 nm and lengths exceeding 50 nm. Raman and XPS analyses confirmed a graphene structure.

### Preparation of GNRs for determining toxicity

To disperse the GNRs particles, we chose a negatively charged TWEEN 20 (Sigma Aldrich, cat. no. P9416), a positively charged cetyltrimethylammonium bromide (CTAB) (Sigma Aldrich, cat. no. H9151), and neutral Triton X-100 (Fisher Scientific, cat. no. BP151), which were prepared by dissolving 0.01 g of each of the surfactants separately into 200 mL of water (0.005% w/v).

**Fig. 1:**
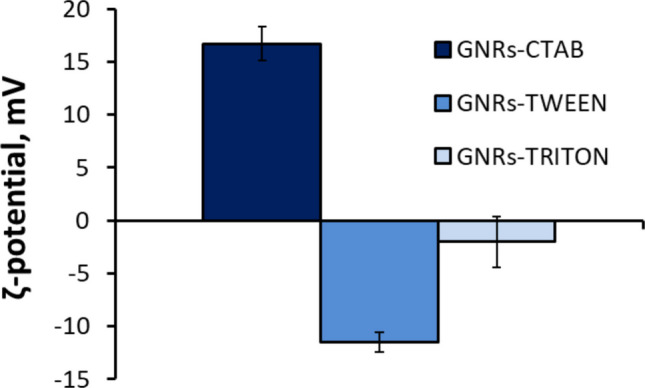
Zeta potential of GNRs particles stabilized in CTAB, TWEEN, and TRITON

The obtained powder of the GNRs particles was dispersed in freshly prepared surfactant solution at a final concentration of 100 mg L^−1^. After, the dispersed surfactant particles were manually ground using a glass pestle for 3 min and then sonicated (37 kHz, 100 W) for 10 min. To reach the best possible distribution of particles, the cycle of sonication and grinding was repeated 4 times. In the end, particles were kept in the same solution containing surfactants. All samples were sterilized by autoclaving at 121 °C for 15 min. Before each experiment, the obtained solution was sonicated (37 kHz, 100 W) for 10 min and diluted to prepare a range of concentrations. The dilutions were made by mixing 100 μL of GNRs particles with 900 μL of MQ to obtain a series of tenfold dilutions from 100 mg L^−1^ to 0.1 mg L^−1^. To ensure the reproducibility of the GNR dispersions, the protocol for surfactant addition, grinding, sonication, and sterilization was standardized and followed identically for all samples and experimental repeats. The colloidal stability of each freshly prepared and sonicated dispersion was verified before each experiment by measuring the ζ-potential (“Determination of zeta potential of GNRs particles”), which served as a key quality control parameter. Dispersions with ζ-potential values consistent with those reported in Fig. 1 were used for further testing.

### Determination of zeta potential of GNRs particles

To determine Zeta Potential, a total of 200 μL of the dispersed GNRs particles in a surfactant was poured in 2.8 mL of MQ water, mixed by mild vortexing, and then measured using an ELS method (Particle analyzer, Delsa Nano, Beckman Coulter). The value of Zeta Potential was obtained from 3 replicates, where each of the measurements was produced from 15 accumulated values of the separated ELS values. During the measurements, the polarities of the electrodes were set fixed with automatically adjustable voltage.

### Determination of cell viability

In order to exclude the inhibitory effects of surfactants two tests were performed. In the first test, selected bacterial cells were exposed to a nutrient medium containing GNR particles dispersed in surfactants (referred to as continuous exposure). In the second test, the cells were mixed with GNR particles dispersed in surfactants, incubated, and then centrifuged to remove any excess of surfactants (referred to as short exposure). Solutions containing pure surfactants (0.005% and 0.0005% w/v) served as controls in the continuous exposure test. In continuous exposure the MIC approach measuring growth at OD_600nm_ was used. We mixed 90 μL of the solution of GNRs particles of various concentrations (100 mg L^−1^ to 0.1 mg L^−1^), and 90 μL of double strength NB1 medium (2xNB) (Sigma Aldrich), where we also added 20 μL of the bacterial culture (OD_600nm_ 0.1). Based on the literature graphene particles can attach on the cells and therefore we tested in a short exposure experiment how these attached particles contribute to the microbial growth. In the short exposure experiments we firstly exposed the cells to the mixture of GNRs. For exposure we used suspension of 500 μL (OD_600nm_ 0.1) of each tested strain that we mixed with 500 μL of GNRs particles of previously prepared varied concentrations (ranging from 100 mg L^−1^ to 0.1 mg L^−1^). The mixture was vortexed and left to incubate for 5 min. Then, cells were obtained from this mixture by centrifugation for 3 min at 5000 g. The supernatant was discarded and the cells were resuspended in 500 μL of sterile 0.9% NaCl. 20 μL of these cells was added to a well of a 96-well plate containing 180 μL of NB1.

MIC in both continuous and short exposure were determined in 96 microwell plates by measuring OD_600nm_ every 30 min for 24 h at 37 °C with 10 s shaking prior to each measurement (Synergy H4, Microplate reader, BioTek). For the control group, we omitted suspension of GNR particles.

### Microscopy imaging

The interaction of bacterial cells and GNRs particles was visualized by phase contrast microscopy using the inverted fluorescent microscope Zeiss Axio Observer Z1 (Zeiss, Germany) with 40× magnification and the digital CCD camera Orca (model no. C4742-95-12NRB, Hamamatsu, Japan). Poly Dispersity Index (PDI) of aggregates consisting of the cells with GNR particles was calculated as the square of standard deviation divided by the mean aggregate diameter.

### Statistical analysis

For statistical evaluation of data sets, two-tailed t-tests (LibreOffice Calc.) were used. The data sets were obtained from the biological triplicates of OD_600nm_ measured every 30 min for 24 h. After this, the data was analyzed by fitting a parametric growth model to obtain the duration of the lag phase time from the growth curves by PRECOG (Fernandez-Ricaud et al. 2016). Graphs were prepared using LibreOffice Calc. (The Document Foundation) and Inkscape (Free Software Foundation).

## Results

### Zeta potential of GNRs particles

In our research, each type of the surfactants contributed to particle charge, changing it from highly positive to negative (Fig. 1). The zeta potential of GNRs-CTAB dispersed particles was the highest (16.7 ± 1.6 mV), and GNRs-TWEEN the lowest (−11.5 ± 0.94). The GNRs-TRITON showed values close to zero (−2.0 ± 2.4).

### Cell viability under continuous exposure to GNRs

**Fig. 2:**
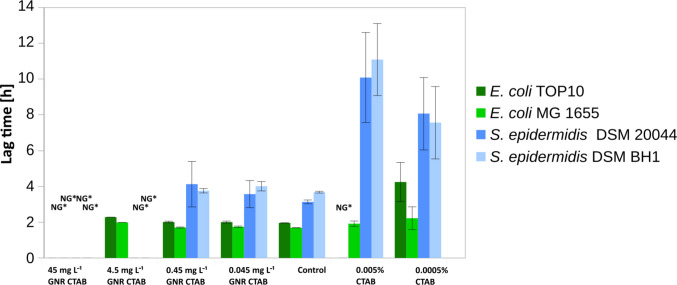
The lag phase time of bacterial cells incubated with positively charged GNR-CTAB in nutrient media over 20 h. Biofilm-forming, biofilm non-forming cells from both Gram+ or Gram− were used to measure bactericidal activity. NG* referred to as no growth was observed

In general, both Gram-positive strains showed approximately 10 times higher sensitivity to the GNR-CTAB, where we could observe the inhibition of growth of *S. epidermidis* 20044 and *S. epidermidis* BH1 strains (Fig. 2). GNR-CTAB particles dispersed in the medium inhibited growth of all bacterial strains at the 45 mg L^−1^ concentration except *S. epidermidis* DSM 20044 and *S. epidermidis* BH1, which were not growing even at 10 times lower concentration (4.5 mg L^−1^) during the 20 h exposure experiment.

In all incubation experiments where bacterial growth was observed at a similar level to the control, the *lag* phase of the cells exposed to GNR-CTAB particles was not significantly different from the control cells (Fig. 2), although the growth curves of both *Escherichia* strains showed a delayed onset of the exponential phase when exposed to 4.5 mg L^−1^ of particles (Fig. S1**)**. In contrast, at this concentration both *Staphylococcus* strains exhibited no growth. Based on this observation, we can conclude that both Gram-positive strains are the most sensitive to GNR-CTAB.

To investigate whether the toxicity of the particles was related to the choice of the stabilizer, we determined the inhibitory action of CTAB dispersed in the medium without GNRs(Fig. 2). In general, the lag phase of Gram-positive bacteria exposed to the lowest concentration of CTAB was 4 times longer than the lag phase of the Gram-negative bacterial strains. Among Gram-negative bacteria, the most sensitive strain was *E. coli* TOP10, which was completely inhibited at the highest concentration, and it showed a 2 times prolonged lag phase at the lowest concentration. The inhibition effect for the *E. coli* MG1655 was not statistically significant although a slight delay in the duration of the lag phase was observed.

Among Gram-positive strains biofilm-forming strain did not show a significant resistance advantage over biofilm non-forming strains and the *lag* phase was significantly prolonged by an average of 9.2 and 7.6 times at the highest and lowest concentrations of CTAB for both strains, respectively (*P* < 0.05).

**Fig. 3:**
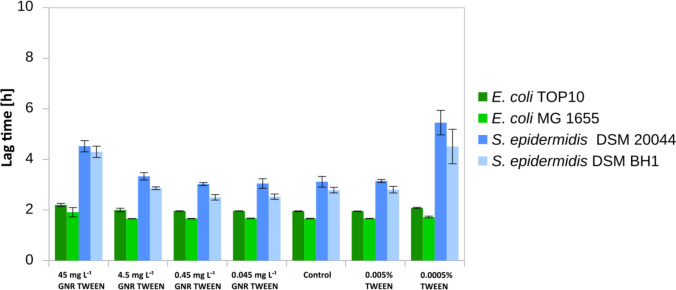
The lag phase time of bacterial cells incubated with negatively charged GNR-TWEEN in nutrient media over 20 h. Biofilm-forming, biofilm non-forming cells from both Gram+ and Gram− were used to measure bactericidal activity

In all strains we observed growth when GNR-TWEEN stabilized particles were added (Fig. 3). Moreover, the highest concentrations of GNR-TWEEN and pure TWEEN stimulated the cell growth (see Fig. S2). Yet, we also observed 1.4 and 1.5 times delayed lag phases for the highest GNR-TWEEN concentration and 1.7 and 1.6 for the lowest concentration of the pure TWEEN for *S. epidermidis* DSM 20044 and *S. epidermidis* BH1 (*P* < 0.05).

**Fig. 4:**
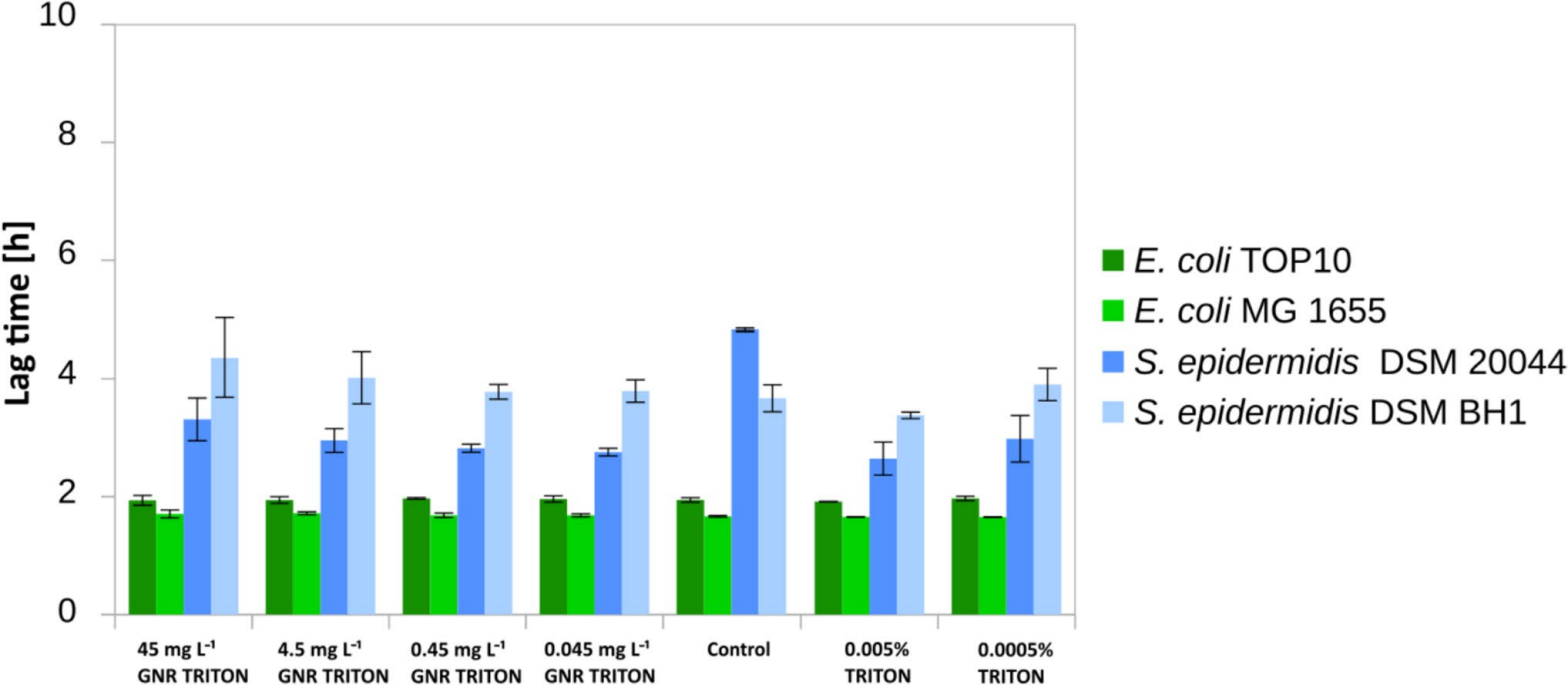
The lag phase time of bacterial cells incubated with neutrally charged GNR-TRITON in nutrient media over 20 h. Biofilm-forming, biofilm non-forming cells from both Gram+ and Gram− were used to measure bactericidal activity

The application of GNR-TRITON stabilized particles in all tested strains did not result in any growth inhibition or extension of the *lag* phase (see Fig. 4) but instead stimulated growth, especially observed among both Gram-positive strains during the exposure to the highest concentrations of GNR-TRITON particles (Fig. S3).

### Cell viability under short exposure to GNRs

**Fig. 5:**
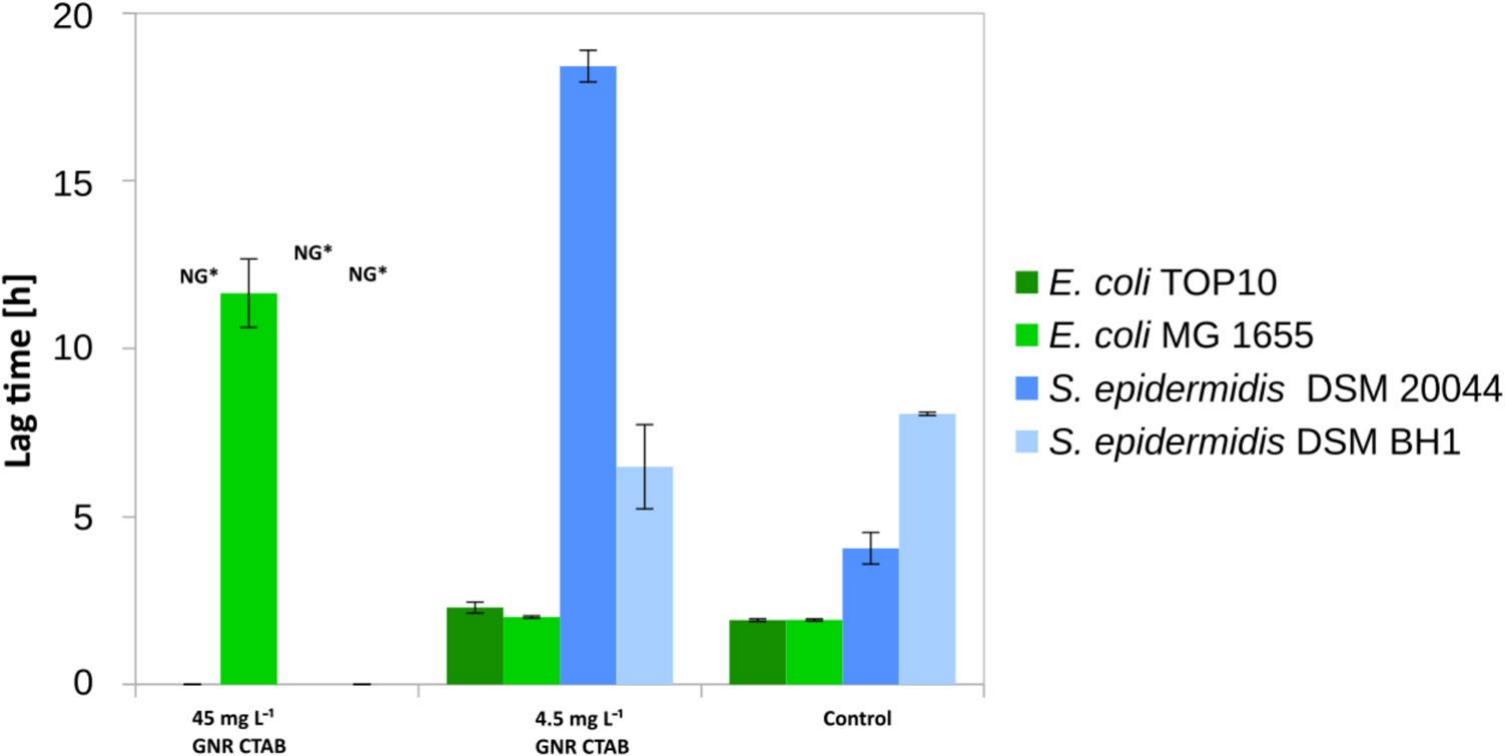
The lag phase time of bacterial cells after short incubation with positively charged GNR-CTAB in nutrient media over 20 h. Biofilm-forming, biofilm non-forming cells from both Gram+ and Gram− were used to measure bactericidal activity

Deposition of GNR-CTAB on the cell surface at the highest concentration inhibited the growth of all tested cells except *E. coli* MG1655, which had a sixfold longer lag phase than control (*P* < 0.01). Instead, a tenfold concentration reduction did not inhibit cell growth for all the strains. The only significantly prolonged lag phases were observed for the biofilm non-forming Gram-negative *E. coli* TOP10 (1.2-fold, *P* < 0.05) and the biofilm-forming Gram-positive *S. epidermidis* DSM20044 (4.5-fold, P < 0.01) (Fig. 5 and S4).

**Fig. 6:**
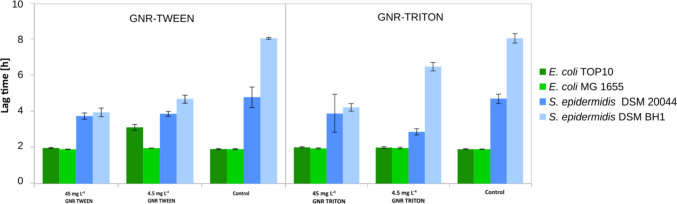
The lag phase time of bacterial cells incubated with negatively and neutrally charged GNR-TWEEN and GNR-TRITON in nutrient media over 20 h. Biofilm-forming, biofilm non-forming cells from both Gram+ and Gram− were used to measure bactericidal activity

Only both biofilm non-forming strains were affected by short exposure to GNRs when stabilized with TWEEN surfactant. The lag phase of *E. coli* Top 10 growth was extended by 1.6 times at the lowest GNR-TWEEN concentration. In contrast, *S. epidermidis* BH1 exhibited a 1.9-fold shorter lag phase for both GNR concentrations than measured in the control samples (*P* < 0.01) (see Fig. 6, S5 and S6).

The *lag* phase of Gram-positive cells exposed to GNR-TRITON was shortened by 1.6 times for biofilm-forming *S. epidermidis* DSM20044 at the lowest concentration and on average by 1.6 times for biofilm non-forming *S. epidermidis* BH1 for both concentrations (*P* < 0.01) (see Fig. 6, S5 and S6).

### Microscopy imaging

**Fig. 7:**
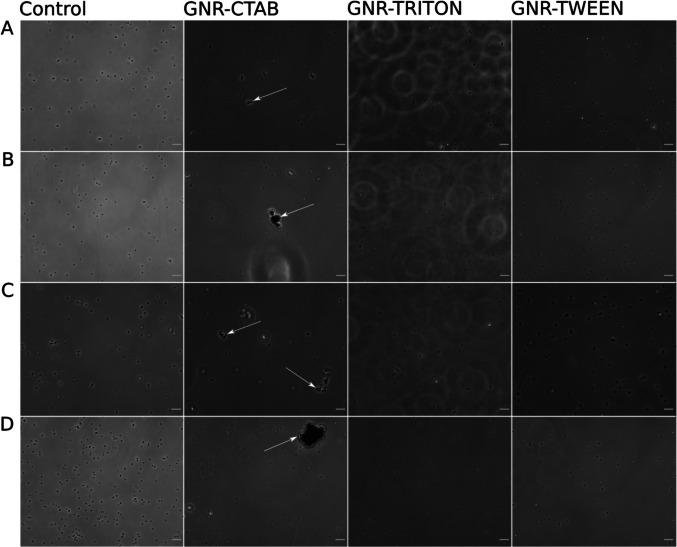
Stabilizers affect aggregation of cells with dispersed GNR particles. **A** *E. coli* TOP10, **B** *S. epidermidis* BH1, **C** *E. coli* MG 1655, **D** *S. epidermidis* 20044. Scale bar = 5 μm

The microscopy analysis showed formation of aggregates of bacterial cells only when exposed to the GNR-CTAB particles (Fig. 7). In particular, the polydispersity index (PDI) was 2.7 times higher for the suspension of biofilm non-forming cells than biofilm-forming (12.2 and 4.6, respectively) when exposed to GNR-CTAB particles. In contrast, in both GNR stabilized with neutral and negative surfactants we could not see any observable effects.

## Discussion

In many applications of GNRs, it requires suspension of particles in aqueous solvents; for instance, in the building industry (Sindu and Sasmal 2017; Piao et al. 2024), thermal conductivity applications of nanofluids (Yu et al. 2011), enhancing polymer mechanical properties (Rafiee et al. 2010), and semiconducting (Li et al. 2008). Therefore, various types of surfactants have been employed to stabilize the suspension. The concentration ranges selected for this study (0.1–100 mg L⁻^1^ for GNRs; 0.0005%–0.005% w/v for surfactants) were chosen to span a broad scope of plausible scenarios. The upper concentrations (tens of mg L⁻^1^) are consistent with doses commonly employed in in vitro studies to evaluate the inherent antibacterial potential and mechanistic pathways of carbon nanomaterials, allowing for direct comparison with existing literature on GO and rGO (e.g., Gusev et al. 2019a; 2019b). The lower end of the concentration approaches levels that could be encountered in specific environmental niches prior to significant dilution, thereby enabling a preliminary assessment of potential environmental hazard. Indeed, modeled environmental concentrations for engineered nanomaterials are typically in the ng L⁻^1^ to μg L⁻^1^ range (Gottschalk et al. 2013); our choice of 0.1 mg L⁻^1^ represents a conservative, higher-tier exposure scenario for hazard identification. Furthermore, surfactant concentrations were deliberately maintained below their critical micelle concentration to isolate their role as colloidal stabilizers from their intrinsic detergent or antimicrobial effects.

According to our study, the choice of surfactant significantly influences the toxicity of the GNR-surfactant combination. Specifically, although cationic surfactants in combination with GNRs exhibited superior properties due to better colloidal stabilization (Nazari et al. 2019) (see Fig. 1), our results showed higher toxic effects on bacteria, with Gram-positive bacterial strains being more susceptible than Gram-negative strains during continuous exposure than when bacteria were exposed to the GNR stabilized with the other two surfactants. This result is challenging to interpret, as existing literature presents conflicting findings regarding the antimicrobial effects of graphene-based nanomaterials. Some studies suggest that Gram-positive bacteria are more sensitive to such materials, while others report the opposite effect (Sengupta et al. 2019). In certain cases, it has been suggested that graphene nanomaterials may even stimulate the growth of bacteria (Palmieri et al. 2017; Wu et al. 2018). Our findings align with this observation in continuous exposure experiments; GNRs combined with neutral and anionic surfactants appeared to stimulate growth, particularly in both *S. epidermidis* strains. In the absence of GNRs, anionic surfactants are known to promote bacterial growth at specific concentrations (Baker et al. 1941). This effect could be attributed to enhanced dispersion of nutrients, especially peptides, present in nutrient-rich media such as NB that was also used in our experiments. The peptone and yeast extract components in the medium may become more accessible to bacterial cells due to the emulsifying properties of the surfactants and decreased surface tension of water. Moreover, the presence of anionic surfactants also increases fluidity of the cell membrane and increases uptake of nutrients (Xiong et al. 2020). The hormetic effect might also contribute to induced growth since small amounts of surfactants induce formation of oxidative stress and better preparedness of cells to the ROS generated by the GNR during the exposure (Ludovico and Burhans 2014). On the other hand, when dispersing GNRs with CTAB, the surface of GNR-CTAB complexes acquired a positive charge (zeta potential 16.7 ± 1.6 mV, see Fig. 1). It is known that positively charged nanoparticles exhibit higher toxicity than negative or neutral charges. The high toxicity of positively charged nanoparticles is attributed to their ability to easily penetrate cells, unlike their negatively charged and neutral counterparts. This is due to electrostatic attraction between negatively charged glycoproteins on the cell membrane and positively charged nanoparticles (Hühn et al. 2013; Sukhanova et al. 2018). Additionally, positively charged particles more strongly bind to negatively charged DNA when entering the cell, decreasing the probability of the success of the cell repair systems (Liu et al. 2011).

Moreover, in the CTAB variant, higher zeta potential values possibly indicate greater suspension stability compared to other tested conditions. The relatively high charge on particle surfaces prevents nanoribbon aggregation, which is a factor in increasing nanoparticle toxicity in prokaryotes. While direct visualization (e.g., TEM) of surfactant-coated GNRs was not performed in this study, the colloidal state of the dispersions—a critical factor influencing bioactivity—was quantitatively assessed via ζ-potential measurements (Fig. 1). The high positive ζ-potential of the GNR-CTAB complex (+ 16.7 mV) is a direct indicator of its stable, non-aggregated state in suspension, which is essential for effective interaction with bacterial cells. In contrast, the near-neutral ζ-potential of systems with TWEEN and TRITON suggests poor electrostatic stabilization and a high propensity for aggregation, which correlates with their lack of antimicrobial efficacy and may explain the observed growth stimulation due to reduced bioavailability of the nanoribbons.

In contrast to the eukaryotic cells, the prokaryotic cells cannot internalize larger aggregates through phagocytosis (Albanese and Chan 2011; Chen et al. 2023). Therefore, direct contact between GNRs and the surface of bacterial cells results in bacterial death through the realization of one or several possible scenarios of nanotoxicity such as direct cell membrane damage, oxidative stress, biopolymer degradation, or disruption of cell wall or membrane lipids (Yuan et al. 2019; PJ et al. 2021). In relation to this, the observed tenfold higher susceptibility of Gram-positive bacteria to GNR-CTAB can be attributed to a combination of nanomaterial properties and cell wall architecture. The used GNRs, with their well-defined narrow width (several to 60 nm), significant length (> 50 nm), and single-layer nature (~ 0.3 nm), present a unique set of physical characteristics. Their high aspect ratio and atomically thin, sharp edges are conducive to direct physical interaction with cell surfaces, a mechanism where the morphology and edge structure of graphene materials are key determinants of membrane interaction (Li et al. 2013). For Gram-positive bacteria, which lack an outer lipid membrane and possess a thick but porous peptidoglycan layer, these rigid, sheet-like nanostructures can effectively insert or adhere to the cell wall, potentially causing local stress, disruption, and enhanced delivery of the associated cationic CTAB. In contrast, the outer membrane of Gram-negative bacteria may act as an initial barrier, mitigating direct membrane damage—a differential susceptibility well-documented for other graphene derivatives (Perreault et al. 2015). Thus, it can be expected that the precise “bottom-up” synthesis yielding GNRs with consistent dimensions is a key factor determining their mode of antibacterial action and target specificity.

Moreover, Sengupta et al. (2019) actually reported that the shape of bacteria affects also the toxicity of GO and rGO, where GO destructs bacteria by cell membrane damage through chemical reaction whereas rGO induces mechanical stress and pierces the cell membrane. Shape and type of bacteria act as the controlling factors in determining the bactericidal efficacy of the nanomaterials where GO can wrap spherical Gram-positive cells better than the bacillary Gram-negative and causing mostly mechanical stress and intracellular nutrient depletion due to the diffusion blockage. The rGO is causing mostly prolonged mechanical as well as oxidative stress on bacillary cells due to the larger surface area and higher curvature that is more appropriate for better interaction with the cell wall and membrane. This might be even more pronounced in GNR since they are 2D materials wrapping both types of cells but cannot easily penetrate the outer lipid membrane of Gram-negative bacteria. Hence, at lower GNR concentrations, we observed Gram-negative bacteria to be 10 times more resistant than Gram-positive bacteria. The cell wall structure of Gram-positive and Gram-negative bacteria plays a major role in their susceptibility to nanoparticles since Gram-negative bacteria possess an additional external lipid membrane in contrast to the Gram-positive bacteria (Ahmad et al. 2020).

The formation of smaller aggregates of Gram-negative bacteria (see Fig. 7) perhaps might be as a result of the accumulation of GNR on the larger surface and better stabilization of the colloidal suspension when CTAB was used. In addition, the microscopic analysis revealed the formation of bacterial cell aggregates and GNR-CTAB complexes, with this effect particularly pronounced in biofilm non-forming bacteria. This phenomenon may be associated with the initial stages of biofilm formation in response to environmental stimuli (Kostakioti et al. 2013; Arunasri and Mohan 2019), as well as electrostatic interactions between cells and nanocomplexes (Gusev et al. 2019b) where the inductions of additional extracellular produced layers might contribute to the microbially controlled aggregation resulting in lower PDI (see “Results”) while among biofilm non-forming strains the aggregation might be induced due to the electrostatic interactions between cells, death cells, and GNR resulting in less controlled aggregative behavior.

The formation of aggregates is observed in many bacteria as a response to chemical stress (Trunk et al. 2018). For example, high concentrations of chloroaromatic compounds led to autoaggregation of *P. putida* CP1 cells in culture, whereas lower concentrations during growth did not induce cell aggregation (Farrell and Quilty 2002). Active formation of cellular aggregates also serves as a survival mechanism in *P. aeruginosa* under stress induced by specific surfactants like sodium dodecyl sulfate (Klebensberger et al. 2007). It was reported that aggregation significantly increased the resistance of *S. aureus* cells to quaternary ammonium compounds (Burel et al. 2021), such as CTAB in contrast to the well-dispersed cell population. Microbial aggregation can be mediated through various mechanisms including exopolysaccharides, extracellular DNA (eDNA), or proteins, as well as chemotaxis (Schleheck et al. 2009; Secor et al. 2018, 2022; Tavaddod et al. 2024).

Effect that can contribute to the lower toxicity or even stimulatory effect among the biofilm non-forming strains can be as a result of the depletion of the toxic GNR by their aggregation that is driven by physical forces between nanoribbons themselves in the surrounding environment (Secor et al. 2022). It is likely that in our study, we observed a similar effect especially among the suspension of anionic and neutral surfactants stabilized GNR since they showed zeta potentials closer to the 0 mV (Hunter 2013). For instance, the both highest concentrations of GNR when anionic surfactant was used biofilm non-forming strains showed close to the control or same growth properties at the 4.5 mgL^−1^ in both types of bacteria. These findings are also likely to occur due to the low stability of GNRs in colloids with TWEEN and TRITON, leading to formation of aggregates at high concentrations. Similar phenomena have been previously described by other authors. For instance, studies by Takenaka et al. (2001) and Gurr et al. (2005) on Ag and TiO_2_ nanoparticles, respectively, demonstrated increased toxicity at lower concentrations compared to higher concentrations. This phenomenon is attributed to nanoparticle aggregation in cultures with higher particle concentrations, resulting in reduced overall toxicity (Ahmad et al. 2020).

Certainly, additional studies are needed to better understand the antimicrobial action of GNRs, including the study of toxicity mechanisms and the development of standardized testing methodologies. It is noteworthy that in our study, GNRs were toxic only when positively charged CTAB was used as a stabilizer, which is commonly employed as an antiseptic (Aronson 2014), potentially enhancing the bactericidal action of GNRs (see Figs. 2, 3) resulting in a synergistic nanotoxic effect, while TWEEN and TRITON even promoted growth in some cases.

Building on the need to understand environmental implications, our findings allow us to hypothesize about potential long-term ecological impacts if surfactant-stabilized GNRs were released. The release of CTAB-based formulations could exert a strong selective pressure, potentially suppressing Gram-positive populations and altering microbial community structure, with possible cascading effects on ecosystem functions like organic matter decomposition. In contrast, systems with non-ionic surfactants might lead to more subtle, unpredictable shifts in community dynamics. Therefore, future eco-toxicological assessments should move beyond single-species tests to evaluate the effects on complex microbial communities and ecosystem functions under environmentally relevant conditions, integrating the molecular-level understanding with higher-level ecological consequences.

## Conclusions

GNRs synthesized via a bottom-up method stabilized by CTAB, TWEEN, or TRITON were used to investigate their antibacterial properties against biofilm-forming and biofilm non-forming cells with Gram-negative and Gram-positive architectures. Detergent type played a decisive role in determining the toxicity of GNRs, as CTAB-stabilized GNRs exhibited the strongest antibacterial effects, demonstrating concentration-dependent inhibition of bacterial growth ranging from 0.45 to 45 mg L⁻^1^. Gram-positive bacteria were found to be 10 times more susceptible to GNRs than Gram-negative bacteria, and the ability of biofilm formation did not significantly alter bacterial susceptibility. CTAB alone displayed antibacterial activity, inducing lag phase delays and partial growth suppression, suggesting synergistic contributions of CTAB and GNRs to the observed toxicity effects.

The GNR-TWEEN and GNR-TRITON variants showed diverse effects, ranging from the extension of the lag phase to stimulation of bacterial growth without any observable complete growth inhibition. The observed growth stimulation, particularly for *S. epidermidis* strains, can be interpreted through several non-exclusive mechanisms grounded in nanotoxicology. First, the adsorption of non-ionic (TRITON) or anionic (TWEEN) surfactants onto the GNR surface may effectively passivate the reactive edges and basal planes, shielding bacterial cells from direct physical damage or oxidative stress typically induced by pristine graphene materials. Second, surfactants like TWEEN 20 are known to emulsify hydrophobic components, potentially increasing the bioavailability of nutrients present in the complex growth medium (e.g., peptones, lipids), thereby creating a stimulatory environment. Finally, a low-concentration, sub-inhibitory exposure to nanomaterial stress can sometimes elicit a hormetic response, where mild stress primes cellular defense and repair mechanisms, leading to temporarily accelerated growth upon adaptation (Iavicoli et al. 2018). While the exact contribution of each mechanism requires further study, their consideration is crucial for accurately predicting the environmental behavior of surfactant-stabilized nanomaterials, where growth stimulation could paradoxically enhance biofilm formation or alter microbial community dynamics.

Microscopic analysis revealed cell aggregation and the formation of bacterial-GNR complexes, particularly in biofilm non-forming bacteria. These effects were absent in systems stabilized with TWEEN or TRITON.

Concluding, this study highlights the importance of stabilizer selection in designing antibacterial materials based on GNRs. Positively charged stabilizers, such as CTAB, enhance electrostatic interactions and promote nanotoxicity mechanisms. Future research should aim to develop uniform toxicity assessment mechanisms and further elucidate the molecular and cellular mechanisms underlying the antibacterial effects of GNRs. Specifically, to unravel the mechanistic basis of the observed synergy and selective toxicity, subsequent studies will focus on (i) quantifying oxidative stress response and membrane damage in real-time using fluorescent probes (e.g., DCFH-DA for ROS, propidium iodide for membrane integrity); (ii) visualizing the nano-bio interface with high-resolution microscopy (TEM, AFM) to track the localization and ultrastructural changes induced by GNR-surfactant complexes; and (iii) employing transcriptomic analysis to identify key genetic pathways activated in response to GNR-CTAB exposure, particularly those related to cell wall maintenance, stress response, and biofilm regulation. Such a multi-faceted approach will ensure a comprehensive understanding of both the efficacy and potential environmental implications of surfactant-stabilized GNRs. Ultimately, these mechanistic insights are a prerequisite for predicting and managing the long-term ecological impact of such nanomaterials

## Supplementary Information

Below is the link to the electronic supplementary material.

ESM 1 (DOCX 1.20 MB)

## Data Availability

The original contributions presented in the study are included in the article/Supplementary Material; further inquiries can be directed to the corresponding author.

## References

[CR1] Ahmad N, Abdullah N, Yasin F (2020) Toxicity assessment of reduced graphene oxide and titanium dioxide nanomaterials on gram-positive and gram-negative bacteria under normal laboratory lighting condition. Toxicol Rep 7:693–699 32528857 10.1016/j.toxrep.2020.04.015 PMC7283152

[CR2] Albanese A, Chan WC (2011) Effect of gold nanoparticle aggregation on cell uptake and toxicity. ACS Nano 5:5478–5489 21692495 10.1021/nn2007496

[CR3] Alexander K, Gajghate SS, Katarkar AS, Majumder A, Bhaumik S (2021) Role of nanomaterials and surfactants for the preparation of graphene nanofluid: a review. Mater Today Proc 44:1136–1143

[CR4] Angelini LL, Dos Santos RAC, Fox G, Paruthiyil S, Gozzi K, Shemesh M, Chai Y (2023) Pulcherrimin protects *Bacillus subtilis* against oxidative stress during biofilm development. NPJ Biofilms Microbiomes 9:50 37468524 10.1038/s41522-023-00418-z PMC10356805

[CR5] Ansón-Casaos A, Mis-Fernández R, López-Alled CM, Almendro-López E, Hernández-Ferrer J, González-Domínguez JM, Martínez MT (2015) Transparent conducting films made of different carbon nanotubes, processed carbon nanotubes, and graphene nanoribbons. Chem Eng Sci 138:566–574

[CR6] Aronson JK (2014) Meyler’s Side Effects of Drugs 15E: The International Encyclopedia of Adverse Drug Reactions and Interactions. Newnes

[CR7] Arunasri K, Mohan SV (2019) Biofilms: microbial life on the electrode surface. In: Microbial Electrochemical Technology. Elsevier, pp 295–313

[CR8] Baker Z, Harrison R, Miller BF (1941) Action of synthetic detergents on the metabolism of bacteria. J Exp Med 73:249 19871076 10.1084/jem.73.2.249 PMC2135128

[CR9] Burel C, Dreyfus R, Purevdorj-Gage L (2021) Physical mechanisms driving the reversible aggregation of *Staphylococcus aureus* and response to antimicrobials. Sci Rep 11:15048 34294832 10.1038/s41598-021-94457-1 PMC8298462

[CR10] Chae SH, Lee YH (2014) Carbon nanotubes and graphene towards soft electronics. Nano Converg 1:1–26 28936384 10.1186/s40580-014-0015-5 PMC5591626

[CR11] Chen L, Hernandez Y, Feng X, Müllen K (2012) From nanographene and graphene nanoribbons to graphene sheets: chemical synthesis. Angew Chem Int Ed 51:7640–7654 10.1002/anie.201201084 22777811

[CR12] Chen Y-H, Nguyen D, Brindley S, Ma T, Xia T, Brune J, Brown JM, Tsai CS-J (2023) The dependence of particle size on cell toxicity for modern mining dust. Sci Rep 13:5101 36991007 10.1038/s41598-023-31215-5 PMC10060429

[CR13] Chowdhury SM, Surhland C, Sanchez Z, Chaudhary P, Kumar MS, Lee S, Peña LA, Waring M, Sitharaman B, Naidu M (2015) Graphene nanoribbons as a drug delivery agent for lucanthone mediated therapy of glioblastoma multiforme. Nanomedicine Nanotechnol Biol Med 11:109–118 10.1016/j.nano.2014.08.001 PMC4280300 25131339

[CR14] Dik DA, Fisher JF, Mobashery S (2018) Cell-wall recycling of the gram-negative bacteria and the nexus to antibiotic resistance. Chem Rev 118:5952–5984 29847102 10.1021/acs.chemrev.8b00277 PMC6855303

[CR15] Farrell A, Quilty B (2002) Substrate-dependent autoaggregation of *Pseudomonas putida* CP1 during the degradation of mono-chlorophenols and phenol. J Ind Microbiol Biotechnol 28:316–324 12032804 10.1038/sj/jim/7000249

[CR16] Fernandez-Ricaud L, Kourtchenko O, Zackrisson M, Warringer J, Blomberg A (2016) PRECOG: a tool for automated extraction and visualization of fitness components in microbial growth phenomics. BMC Bioinformatics 17:1–15 27334112 10.1186/s12859-016-1134-2 PMC4917999

[CR17] Foreman H-CC, Lalwani G, Kalra J, Krug LT, Sitharaman B (2017) Gene delivery to mammalian cells using a graphene nanoribbon platform. J Mater Chem B 5:2347–2354 32263626 10.1039/c6tb03010f

[CR18] Frontiñan-Rubio J, González VJ, Vázquez E, Durán-Prado M (2022) Rapid and efficient testing of the toxicity of graphene-related materials in primary human lung cells. Sci Rep 12:7664 35538131 10.1038/s41598-022-11840-2 PMC9088729

[CR19] Fu X, Xu L, Li J, Sun X, Peng H (2018) Flexible solar cells based on carbon nanomaterials. Carbon 139:1063–1073

[CR20] Gottschalk F, Sun T, Nowack B (2013) Environmental concentrations of engineered nanomaterials: review of modeling and analytical studies. Environ Pollut 181:287–300 23856352 10.1016/j.envpol.2013.06.003

[CR21] Gungordu Er S, Edirisinghe M, Tabish TA (2023) Graphene‐based nanocomposites as antibacterial, antiviral and antifungal agents. Adv Healthc Mater 12:2201523 36511355 10.1002/adhm.202201523 PMC11468666

[CR22] Gurr J-R, Wang AS, Chen C-H, Jan K-Y (2005) Ultrafine titanium dioxide particles in the absence of photoactivation can induce oxidative damage to human bronchial epithelial cells. Toxicology 213:66–73 15970370 10.1016/j.tox.2005.05.007

[CR23] Gusev A, Zakharova O, Muratov DS, Vorobeva NS, Sarker M, Rybkin I, Bratashov D, Kolesnikov E, Lapanje A, Kuznetsov DV (2019a) Medium-dependent antibacterial properties and bacterial filtration ability of reduced graphene oxide. Nanomaterials 9:1454 31614934 10.3390/nano9101454 PMC6835404

[CR24] Gusev A, Zakharova O, Vasyukova I, Muratov DS, Rybkin I, Bratashov D, Lapanje A, Il’inikh I, Kolesnikov E, Kuznetsov D (2019b) Effect of GO on bacterial cells: role of the medium type and electrostatic interactions. Mater Sci Eng C Mater Biol Appl 99:275–281 30889701 10.1016/j.msec.2019.01.093

[CR25] Hühn D, Kantner K, Geidel C, Brandholt S, De Cock I, Soenen SJ, Rivera_Gil P, Montenegro J-M, Braeckmans K, Mullen K (2013) Polymer-coated nanoparticles interacting with proteins and cells: focusing on the sign of the net charge. ACS Nano 7:3253–3263 23566380 10.1021/nn3059295

[CR26] Hui L, Piao J-G, Auletta J, Hu K, Zhu Y, Meyer T, Liu H, Yang L (2014) Availability of the basal planes of graphene oxide determines whether it is antibacterial. ACS Appl Mater Interfaces 6:13183–13190 25026597 10.1021/am503070z

[CR27] Hunter RJ (2013) Zeta potential in colloid science: principles and applications. Academic press

[CR28] Iavicoli I, Leso V, Fontana L, Calabrese EJ (2018) Nanoparticle exposure and hormetic dose–responses: an update. Int J Mol Sci 19:805 29534471 10.3390/ijms19030805 PMC5877666

[CR29] Jarosz A, Skoda M, Dudek I, Szukiewicz D (2016) Oxidative stress and mitochondrial activation as the main mechanisms underlying graphene toxicity against human cancer cells. Oxid Med Cell Longev 2016:5851035 26649139 10.1155/2016/5851035 PMC4662972

[CR30] Jc PJ, Saigeetha S, Samrot AV, Ponniah P, Chakravarthi S (2021) Overview on toxicity of nanoparticles, it’s mechanism, models used in toxicity studies and disposal methods–a review. Biocatal Agric Biotechnol 36:102117

[CR31] Kim WS, Kim YI, Kim HJ, Hwanag JY, Moon SY, Park N-H, Shim KB, Kim HW, Ham H, Huh H (2011) Fabrication of a large scale transparent conducting film using transformed few-layered graphene nanoribbons obtained from unzipping of single wall carbon nanotubes. J Mater Chem A 21:15655–15659

[CR32] Klebensberger J, Lautenschlager K, Bressler D, Wingender J, Philipp B (2007) Detergent-induced cell aggregation in subpopulations of *Pseudomonas aeruginosa* as a preadaptive survival strategy. Environ Microbiol 9:2247–2259 17686022 10.1111/j.1462-2920.2007.01339.x

[CR33] Kostakioti M, Hadjifrangiskou M, Hultgren SJ (2013) Bacterial biofilms: development, dispersal, and therapeutic strategies in the dawn of the postantibiotic era. Cold Spring Harb Perspect Med 3:a010306 23545571 10.1101/cshperspect.a010306 PMC3683961

[CR34] Lekmeechai S, Su Y-C, Brant M, Alvarado-Kristensson M, Vallström A, Obi I, Arnqvist A, Riesbeck K (2018) *Helicobacter pylori* outer membrane vesicles protect the pathogen from reactive oxygen species of the respiratory burst. Front Microbiol 9:1837 30245670 10.3389/fmicb.2018.01837 PMC6137165

[CR35] Li X, Wang X, Zhang L, Lee S, Dai H (2008) Chemically derived, ultrasmooth graphene nanoribbon semiconductors. Science 319:1229–1232 18218865 10.1126/science.1150878

[CR36] Li Y, Yuan H, Von Dem Bussche A, Creighton M, Hurt RH, Kane AB, Gao H (2013) Graphene microsheets enter cells through spontaneous membrane penetration at edge asperities and corner sites. Proc Natl Acad Sci U S A 110:12295–12300 23840061 10.1073/pnas.1222276110 PMC3725082

[CR37] Liu Y, Li W, Lao F, Liu Y, Wang L, Bai R, Zhao Y, Chen C (2011) Intracellular dynamics of cationic and anionic polystyrene nanoparticles without direct interaction with mitotic spindle and chromosomes. Biomaterials 32:8291–8303 21810539 10.1016/j.biomaterials.2011.07.037

[CR38] Ludovico P, Burhans WC (2014) Reactive oxygen species, ageing and the hormesis police. FEMS Yeast Res 14:33–39 23965186 10.1111/1567-1364.12070 PMC4332618

[CR39] Lushchak V (2001) Oxidative stress and mechanisms of protection against it in bacteria. Biochem Mosc 66:476–489 10.1023/a:1010294415625 11405881

[CR40] Ma J, Feng X (2024) Chain-growth polymerization enables the controlled synthesis of graphene nanoribbons. Chem 10:435–437

[CR41] Majumder P, Gangopadhyay R (2022) Evolution of graphene oxide (GO)-based nanohybrid materials with diverse compositions: an overview. RSC Adv 12:5686–5719 35425552 10.1039/d1ra06731a PMC8981679

[CR42] Miao W, Wang L, Mu X, Wang J (2021) The magical photoelectric and optoelectronic properties of graphene nanoribbons and their applications. J Mater Chem C 9:13600–13616

[CR43] Mohammed H, Kumar A, Bekyarova E, Al-Hadeethi Y, Zhang X, Chen M, Ansari MS, Cochis A, Rimondini L (2020) Antimicrobial mechanisms and effectiveness of graphene and graphene-functionalized biomaterials. A scope review. Front Bioeng Biotechnol 8:465 32523939 10.3389/fbioe.2020.00465 PMC7261933

[CR44] Narita A, Feng X, Müllen K (2015) Bottom-up synthesis of chemically precise graphene nanoribbons. Chem Rec 15:295–309 25414146 10.1002/tcr.201402082

[CR45] Nazari B, Ranjbar Z, Moghaddam AR, Momen G, Ranjbar B (2019) Dispersing graphene in aqueous media: investigating the effect of different surfactants. Colloids Surf A Physicochem Eng Asp 582:123870

[CR46] Nikolic P, Mudgil P (2023) The cell wall, cell membrane and virulence factors of *Staphylococcus aureus* and their role in antibiotic resistance. Microorganisms 11:259 36838224 10.3390/microorganisms11020259 PMC9965861

[CR47] Palmieri V, Bugli F, Lauriola MC, Cacaci M, Torelli R, Ciasca G, Conti C, Sanguinetti M, Papi M, De Spirito M (2017) Bacteria meet graphene: modulation of graphene oxide nanosheet interaction with human pathogens for effective antimicrobial therapy. ACS Biomater Sci Eng 3:619–627 33429629 10.1021/acsbiomaterials.6b00812

[CR48] Paulechka E, Wassenaar TA, Kroenlein K, Kazakov A, Smolyanitsky A (2016) Nucleobase-functionalized graphene nanoribbons for accurate high-speed DNA sequencing. Nanoscale 8:1861–1867 26731166 10.1039/c5nr07061a

[CR49] Peng L-M, Zhang Z, Qiu C (2019) Carbon nanotube digital electronics. Nat Electron 2:499–505

[CR50] Perreault F, De Faria AF, Nejati S, Elimelech M (2015) Antimicrobial properties of graphene oxide nanosheets: why size matters. ACS Nano 9:7226–7236 26091689 10.1021/acsnano.5b02067

[CR51] Piao T, Li P, Im S, Liu J, Choi H, Bae S (2024) Impacts of graphene nanoribbon dispersion and stability on the mechanical and hydration properties of cement paste: insights from surfactant-assisted ultrasonication. J Build Eng 96:110469

[CR52] Pulingam T, Thong KL, Appaturi JN, Lai CW, Leo BF (2021) Mechanistic actions and contributing factors affecting the antibacterial property and cytotoxicity of graphene oxide. Chemosphere 281:130739 34004516 10.1016/j.chemosphere.2021.130739

[CR53] Qiang S, Li Z, Zhang L, Luo D, Geng R, Zeng X, Liang J, Li P, Fan Q (2021) Cytotoxic effect of graphene oxide nanoribbons on *Escherichia coli*. Nanomaterials 11:1339 34069641 10.3390/nano11051339 PMC8160729

[CR54] Rafiee MA, Lu W, Thomas AV, Zandiatashbar A, Rafiee J, Tour JM, Koratkar NA (2010) Graphene nanoribbon composites. ACS Nano 4:7415–7420 21080652 10.1021/nn102529n

[CR55] Rastogi N, Frehel C, Ryter A, Ohayon H, Lesourd M, David HL (1981) Multiple drug resistance in *Mycobacterium avium*: is the wall architecture responsible for exclusion of antimicrobial agents? Antimicrob Agents Chemother 20:666–677 6798925 10.1128/aac.20.5.666 PMC181770

[CR56] Ricci R, Leite N, Da-Silva N, Pacheco-Soares C, Canevari R, Marciano F, Webster T, Lobo A (2017) Graphene oxide nanoribbons as nanomaterial for bone regeneration: effects on cytotoxicity, gene expression and bactericidal effect. Mater Sci Eng C Mater Biol 78:341–348 10.1016/j.msec.2017.03.278 28575993

[CR57] Schleheck D, Barraud N, Klebensberger J, Webb JS, McDougald D, Rice SA, Kjelleberg S (2009) *Pseudomonas aeruginosa* PAO1 preferentially grows as aggregates in liquid batch cultures and disperses upon starvation. PLoS ONE 4:e5513 19436737 10.1371/journal.pone.0005513 PMC2677461

[CR58] Secor PR, Michaels LA, Ratjen A, Jennings LK, Singh PK (2018) Entropically driven aggregation of bacteria by host polymers promotes antibiotic tolerance in *Pseudomonas aeruginosa*. Proc Natl Acad Sci USA 115:10780–10785 30275316 10.1073/pnas.1806005115 PMC6196481

[CR59] Secor PR, Michaels LA, Bublitz DC, Jennings LK, Singh PK (2022) The depletion mechanism actuates bacterial aggregation by exopolysaccharides and determines species distribution & composition in bacterial aggregates. Front Cell Infect Microbiol 12:869736 35782109 10.3389/fcimb.2022.869736 PMC9243289

[CR60] Sengupta J (2020) Application of carbon nanomaterials in the electronic industry. In: Handbook of Nanomaterials for Manufacturing Applications. Elsevier, pp 421–450

[CR61] Sengupta I, Bhattacharya P, Talukdar M, Neogi S, Pal SK, Chakraborty S (2019) Bactericidal effect of graphene oxide and reduced graphene oxide: influence of shape of bacteria. Colloid Interface Sci Commun 28:60–68

[CR62] Shekhirev M, Lipatov A, Torres A, Vorobeva NS, Harkleroad A, Lashkov A, Sysoev V, Sinitskii A (2020) Highly selective gas sensors based on graphene nanoribbons grown by chemical vapor deposition. ACS Appl Mater Interfaces 12:7392–7402 32011111 10.1021/acsami.9b13946

[CR63] Silva M, Caridade SG, Vale AC, Cunha E, Sousa MP, Mano JF, Paiva MC, Alves NM (2017) Biomedical films of graphene nanoribbons and nanoflakes with natural polymers. RSC Adv 7:27578–27594

[CR64] Sindu B, Sasmal S (2017) Properties of carbon nanotube reinforced cement composite synthesized using different types of surfactants. Constr Build Mater 155:389–399

[CR65] Stewart PS (2002) Mechanisms of antibiotic resistance in bacterial biofilms. Int J Med Microbiol 292:107–113 12195733 10.1078/1438-4221-00196

[CR66] Storz G, Tartaglia LA, Farr SB, Ames BN (1990) Bacterial defenses against oxidative stress. Trends Genet 6:363–368 1965068 10.1016/0168-9525(90)90278-e

[CR67] Sukhanova A, Bozrova S, Sokolov P, Berestovoy M, Karaulov A, Nabiev I (2018) Dependence of nanoparticle toxicity on their physical and chemical properties. Nanoscale Res Lett 13:1–21 29417375 10.1186/s11671-018-2457-x PMC5803171

[CR68] Sun K, Li X, Chen L, Zhang H, Chi L (2020) Substrate-controlled synthesis of 5-armchair graphene nanoribbons. J Phys Chem C 124:11422–11427

[CR69] Takenaka S, Karg E, Roth C, Schulz H, Ziesenis A, Heinzmann U, Schramel P, Heyder J (2001) Pulmonary and systemic distribution of inhaled ultrafine silver particles in rats. Environ Health Perspect 109:547–551 10.1289/ehp.01109s4547 PMC1240579 11544161

[CR70] Tavaddod S, Dawson A, Allen RJ (2024) Bacterial aggregation triggered by low-level antibiotic-mediated lysis. Npj Biofilms Microbiomes 10:90 39327479 10.1038/s41522-024-00553-1 PMC11427687

[CR71] Trunk T, Khalil HS, Leo JC (2018) Bacterial autoaggregation. AIMS Microbiol 4:140 31294207 10.3934/microbiol.2018.1.140 PMC6605025

[CR72] Vasconcellos LMR, Santana-Melo GF, Silva E, Pereira VF, Araújo JCR, Silva ADR, Furtado AS, Elias CdeMV, Viana BC, Marciano FR (2021) Electrospun poly (butylene-adipate-co-terephthalate)/nano-hydroxyapatite/graphene nanoribbon scaffolds improved the in vivo osteogenesis of the neoformed bone. J Funct Biomater 12:11 33562592 10.3390/jfb12010011 PMC7931057

[CR73] Vo TH, Shekhirev M, Kunkel DA, Orange F, Guinel MJ-F, Enders A, Sinitskii A (2014) Bottom-up solution synthesis of narrow nitrogen-doped graphene nanoribbons. Chem Commun 50:4172–4174 10.1039/c4cc00885e 24623056

[CR74] Wieland L, Li H, Rust C, Chen J, Flavel BS (2021) Carbon nanotubes for photovoltaics: from lab to industry. Adv Energy Mater 11:2002880

[CR75] Wu P-C, Chen H-H, Chen S-Y, Wang W-L, Yang K-L, Huang C-H, Kao H-F, Chang J-C, Hsu C-LL, Wang J-Y (2018) Graphene oxide conjugated with polymers: a study of culture condition to determine whether a bacterial growth stimulant or an antimicrobial agent? J Nanobiotechnology 16:1–20 29321058 10.1186/s12951-017-0328-8 PMC5761102

[CR76] Xiong H, Liu Y, Xu Q (2020) Effect of sodium dodecyl sulfate on the production of L-isoleucine by the fermentation of *Corynebacterium glutamicum*. Bioengineered 11:1124–1136 33084479 10.1080/21655979.2020.1831364 PMC8291810

[CR77] Yu W, Xie H, Wang X, Wang X (2011) Significant thermal conductivity enhancement for nanofluids containing graphene nanosheets. Phys Lett A 375:1323–1328

[CR78] Yuan X, Zhang X, Sun L, Wei Y, Wei X (2019) Cellular toxicity and immunological effects of carbon-based nanomaterials. Part Fibre Toxicol 16:1–27 30975174 10.1186/s12989-019-0299-z PMC6460856

[CR79] Zakharova OV, Mastalygina EE, Golokhvast KS, Gusev AA (2021) Graphene nanoribbons: prospects of application in biomedicine and toxicity. Nanomaterials 11:2425 34578739 10.3390/nano11092425 PMC8469389

[CR80] Zaytseva O, Neumann G (2016) Carbon nanomaterials: production, impact on plant development, agricultural and environmental applications. Chem Biol Technol Agric 3:1–26

[CR81] Zhang J, Huang L, Zhang Y, Xue Y, Zhang E, Wang H, Kong Z, Xi J, Ji Z (2015) Controlled synthesis of graphene nanoribbons for field effect transistors. J Alloys Compd 649:933–938

[CR82] Zhang M, Yu Q, Liang C, Liu Z, Zhang B, Li M (2016) Graphene oxide induces plasma membrane damage, reactive oxygen species accumulation and fatty acid profiles change in *Pichia pastoris*. Ecotoxicol Environ Saf 132:372–378 27376352 10.1016/j.ecoenv.2016.06.031

[CR83] Zou X, Zhang L, Wang Z, Luo Y (2016) Mechanisms of the antimicrobial activities of graphene materials. J Am Chem Soc 138:2064–2077 26824139 10.1021/jacs.5b11411

