# Supplementary material for: Functional synergistic effects of graphene nanoribbons and surfactant stabilizers on inhibition of growth of biofilm-forming and biofilm non-forming bacteria
Source: Environ Sci Pollut Res Int. 2026 Mar 27;33(13):5965–77. doi: 10.1007/s11356-026-37570-w (PMC13095989; doi:10.1007/s11356-026-37570-w)
Supplement: Supplementary file 1 — (DOCX 1.20 MB) [file 11356_2026_37570_MOESM1_ESM.docx]

**Title: Functional Synergistic Effects of Graphene Nanoribbons and Surfactant Stabilizers on Inhibition of Growth of Biofilm-Forming and Biofilm Non-Forming Bacteria**

**Authors**

Iaroslav Rybkin^1^, Olga Zakharova^2,3^, Alexander Gusev^2,3^, Ales Lapanje^1^

**Affiliations**

^1^ Jozef Stefan Institute, Department on Environmental Sciences, Colloid Biology Laboratory, Ljubljana 1000, Slovenia

^2^ Research Institute for Environmental Science and Biotechnology, Derzhavin Tambov State University, Tambov 392000, Russia

^3^ National University of Science and Technology MISIS, Moscow 119991, Russia

~~
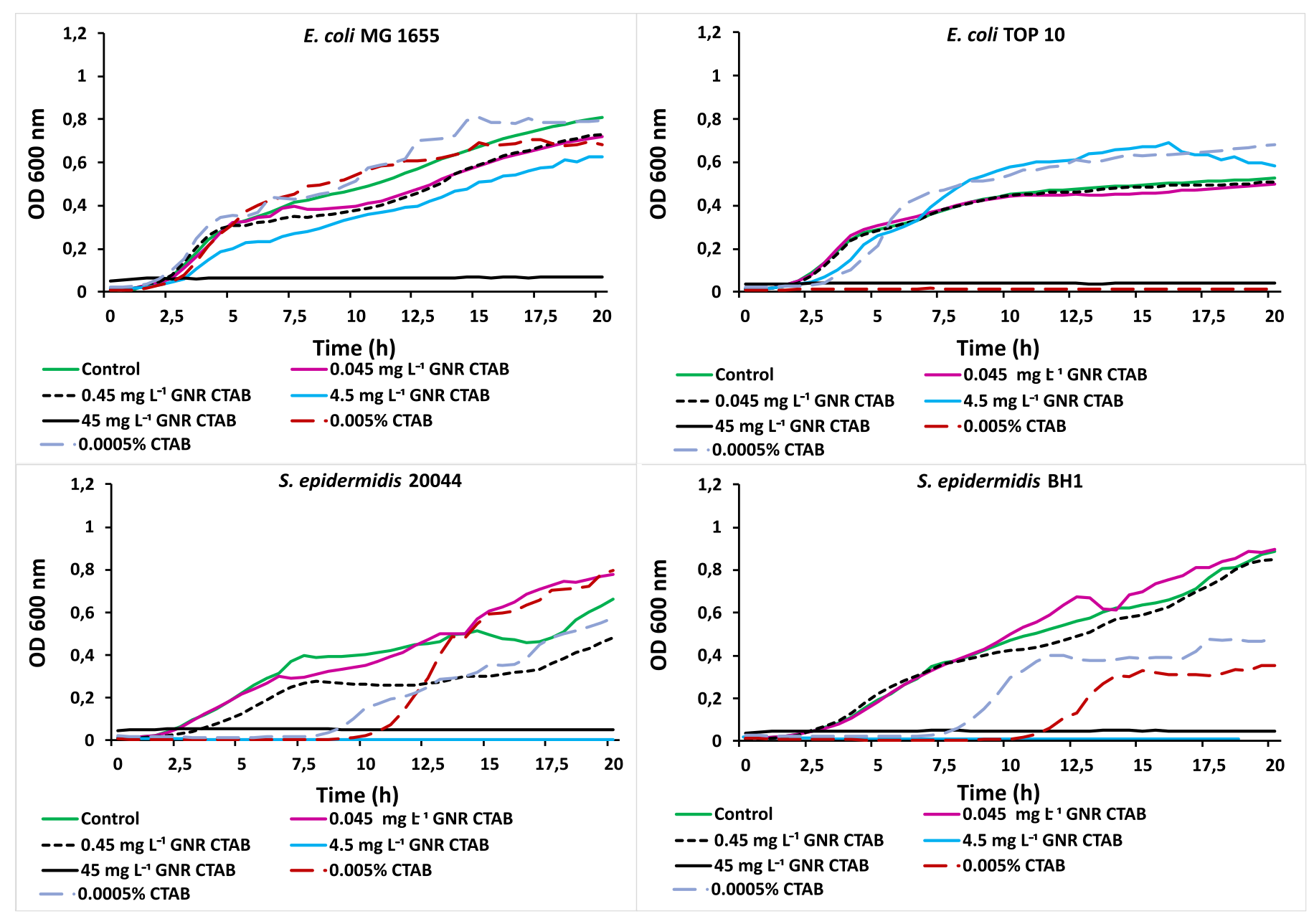
~~

**Fig. S1** Growth of bacterial cells incubated with positively charged GNRs – CTAB in nutrient media over 20 h. Biofilm-forming and biofilm non-forming cells from both Gram + or Gram – were used to measure bactericidal activity.


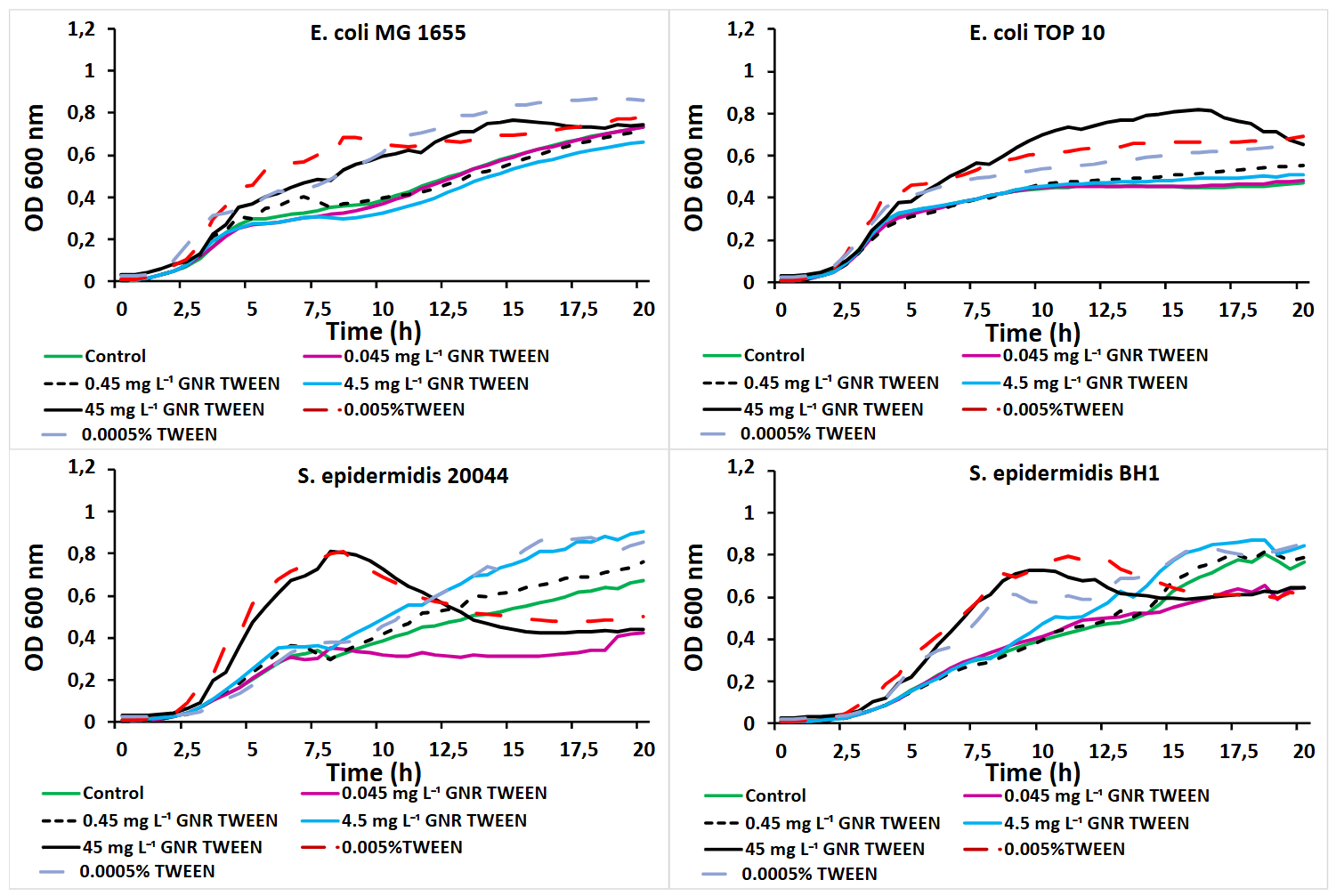


**Fig. S2** Growth of bacterial cells incubated with negatively charged GNRs – TWEEN in nutrient media over 20 h. Biofilm-forming and biofilm non-forming cells from both Gram + or Gram – were used to measure bactericidal activity.


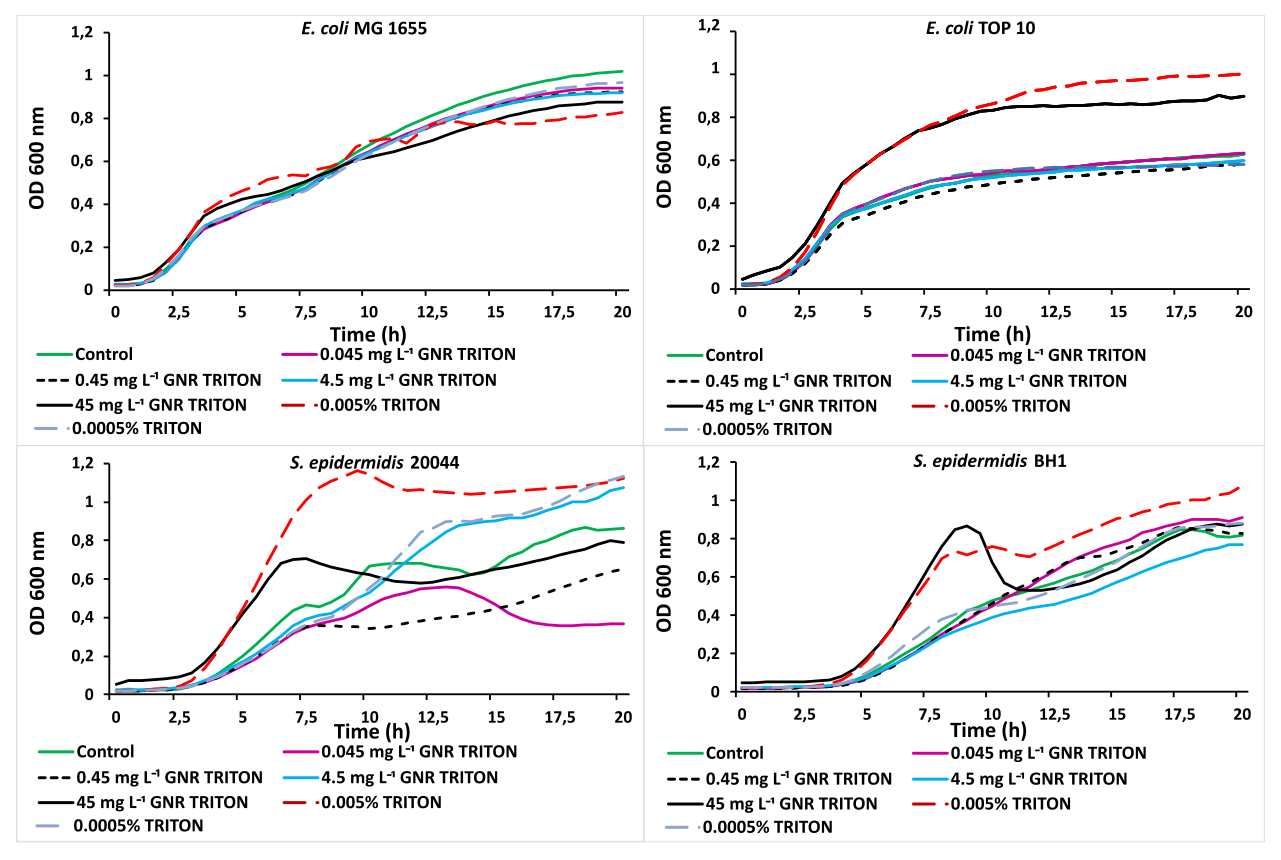


**Fig. S3** Growth of bacterial cells incubated with neutrally charged GNRs – TRITON in nutrient media over 20 h. Biofilm-forming and biofilm non-forming cells from both Gram + or Gram – were used to measure bactericidal activity.


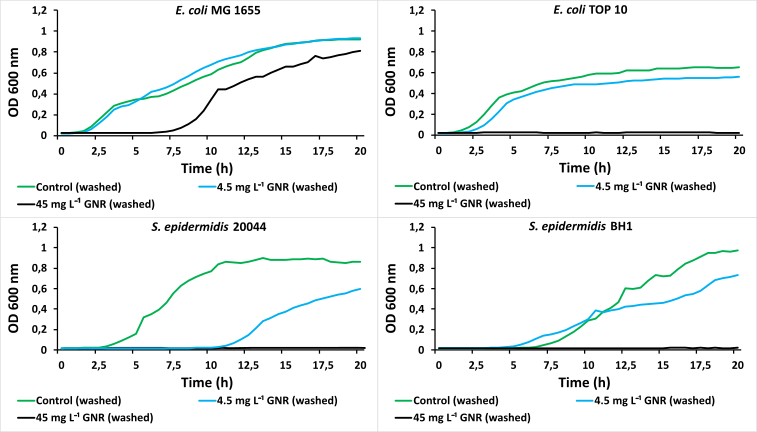


**Fig. S4** Bactericidal effect of short exposure of positively charged CTAB stabilizer on growth biofilm-forming and biofilm non-forming cells from both Gram + or Gram-. The growth of bacteria was examined over 20 h in nutrient broth medium.


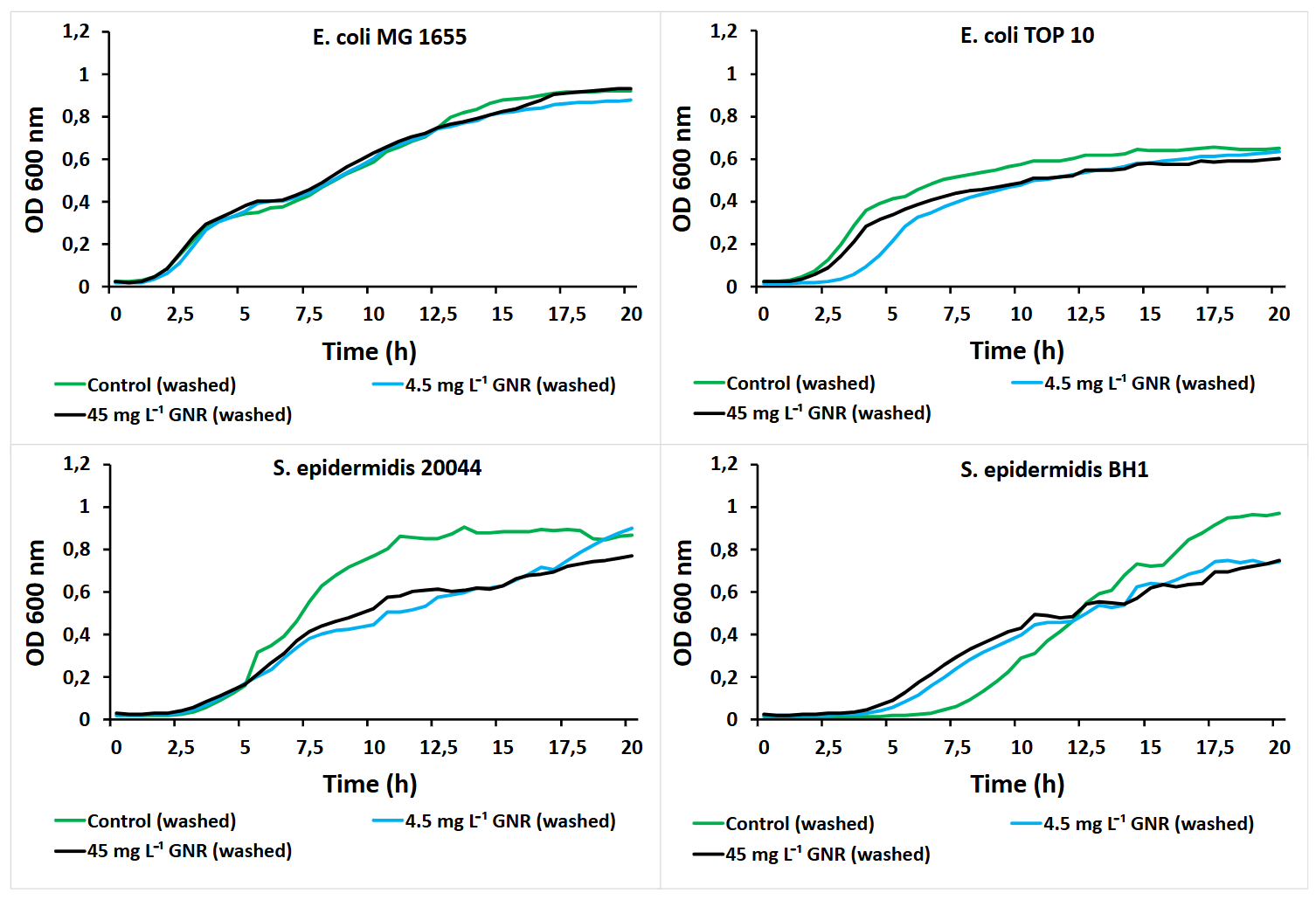


**Fig. S5** Effect of short exposure of negatively charged TWEEN stabilizer on growth biofilm-forming and biofilm non-forming cells from both Gram + or Gram-. The growth of bacteria was examined over 20 h in nutrient broth medium.


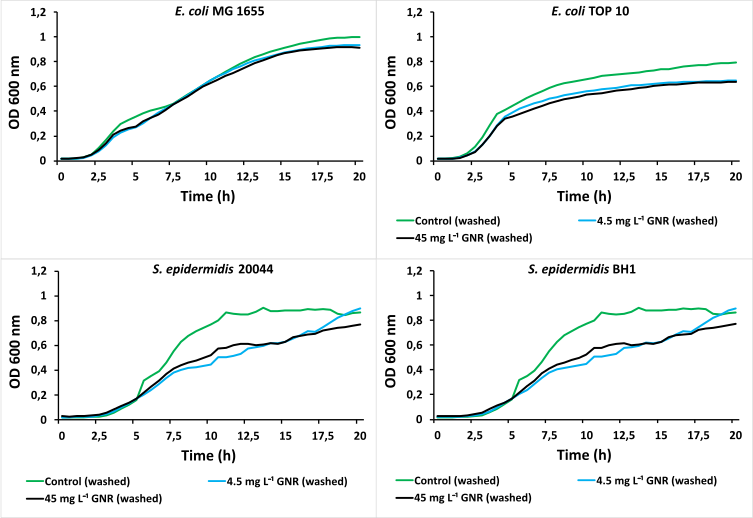


**Fig. S6** Effect of short exposure of neutrally charged TRITON stabilizer on growth biofilm-forming and biofilm non-forming cells from both Gram + or Gram-. The growth of bacteria was examined over 20 h in nutrient broth medium.
